# Diatom-Specific Highly Branched Isoprenoids as Biomarkers in Antarctic Consumers

**DOI:** 10.1371/journal.pone.0056504

**Published:** 2013-02-13

**Authors:** Aurélie Goutte, Yves Cherel, Marie-Noëlle Houssais, Vincent Klein, Catherine Ozouf-Costaz, Mireille Raccurt, Camille Robineau, Guillaume Massé

**Affiliations:** 1 Laboratoire d'océanographie et du climat: expérimentations et approches numériques, UMR 7159 CNRS, Université Pierre et Marie Curie, Paris, France; 2 Centre d'Etudes Biologiques de Chizé, UPR 1934 CNRS, Beauvoir sur Niort, France; 3 Muséum National d'Histoire Naturelle, UMR 7138 CNRS, Paris, France; 4 Université de Lyon; UMR 5023 CNRS, Villeurbanne, France; 5 CNRS & Université Laval, UMI 3376, Takuvik, Québec, Canada; Phillip Island Nature Parks, Australia

## Abstract

The structure, functioning and dynamics of polar marine ecosystems are strongly influenced by the extent of sea ice. Ice algae and pelagic phytoplankton represent the primary sources of nutrition for higher trophic-level organisms in seasonally ice-covered areas, but their relative contributions to polar marine consumers remain largely unexplored. Here, we investigated the potential of diatom-specific lipid markers and highly branched isoprenoids (HBIs) for estimating the importance of these two carbon pools in an Antarctic pelagic ecosystem. Using GC-MS analysis, we studied HBI biomarkers in key marine species over three years in Adélie Land, Antarctica: euphausiids (ice krill *Euphausia crystallorophias* and Antarctic krill *E. superba*), fish (bald notothens *Pagothenia borchgrevinki* and Antarctic silverfish *Pleuragramma antarcticum*) and seabirds (Adélie penguins *Pygoscelis adeliae*, snow petrels *Pagodroma nivea* and cape petrels *Daption capense*). This study provides the first evidence of the incorporation of HBI lipids in Antarctic pelagic consumers. Specifically, a di-unsaturated HBI (diene) of sea ice origin was more abundant in ice-associated species than in pelagic species, whereas a tri-unsaturated HBI (triene) of phytoplanktonic origin was more abundant in pelagic species than in ice-associated species. Moreover, the relative abundances of diene and triene in seabird tissues and eggs were higher during a year of good sea ice conditions than in a year of poor ice conditions. In turn, the higher contribution of ice algal derived organic matter to the diet of seabirds was related to earlier breeding and higher breeding success. HBI biomarkers are a promising tool for estimating the contribution of organic matter derived from ice algae in pelagic consumers from Antarctica.

## Introduction

Shrinking sea ice threatens the structure, functioning and dynamics of polar marine ecosystems [1], [2], [3]. Many species rely on sea ice to complete their life history. At the base of the polar marine trophic web, some micro-algae, primarily pennate diatoms, bloom under and within sea ice in the spring [4], [5], [6]. A second source of primary production is pelagic phytoplankton, which bloom during the summer ice melts. Sea ice decay and breakup modifies the physical and chemical parameters of oceans, including water column stability, nutrient availability, salinity and solar radiation [7], [8]. Several attempts have been made to estimate the relative contribution of ice algae and phytoplankton to primary production in Arctic and Antarctic waters [5], [9]. On an annual basis, ice-related primary production contributes approximately 5 to 28% of the total production in the ice-covered waters of the Southern Ocean [5], [6]. Although ice algae are a significant (direct or indirect) source of nutrition for zooplankton, krill, fish, seabirds and marine mammals [6], their relative contribution to polar marine ecosystems remains poorly understood. Recently, a method to trace carbon flow through Arctic marine trophic webs by analysing the stable isotopes of fatty acids was proposed [10]. Although elegant, the method presents some limitations because of the isotopic fractionation of some fatty acids during ingestion [11].

**Figure 1:**
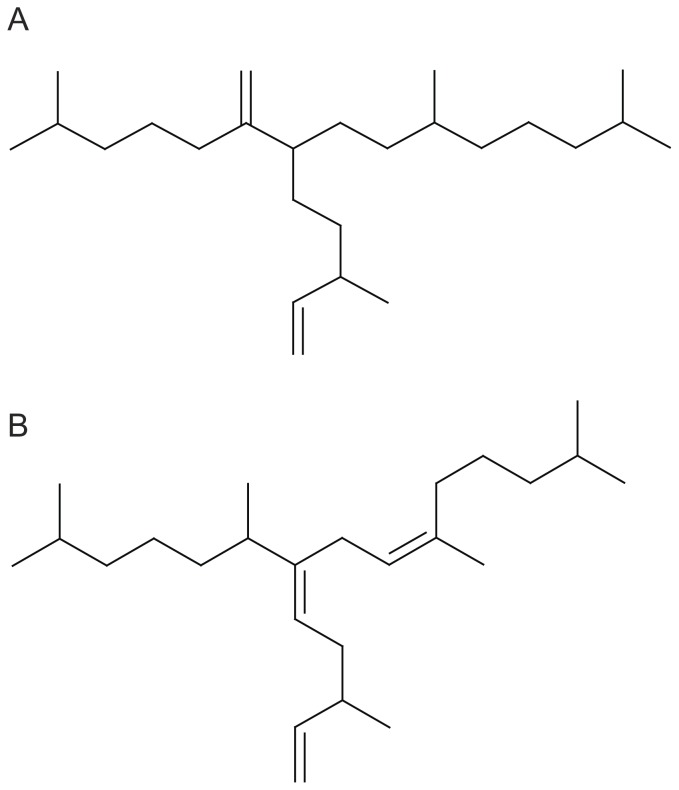
Structure of the two HBIs biomarkers: (A) diene and (B) triene.

The aim of the present study was to evaluate the potential of some novel lipid markers, highly branched isoprenoids (HBIs), for estimating the contribution of organic matter derived from ice algae in pelagic consumers from Antarctica. C_25_ HBIs are synthesised by a few diatom species [12]. In Antarctica, a di-unsaturated HBI isomer (diene, Figure 1 A) was identified in lipid fractions from diatom communities in sea ice, whereas other HBIs, such as a tri-unsaturated isomer (triene, Figure 1 B), were absent in sea ice samples but detected in ice edge and open ocean phytoplankton communities [13], [14]. Recent investigations into the carbon isotopic composition of diene isolated from Antarctic sea ice and sediments revealed that this isomer was isotopically ^13^C enriched relative to the more unsaturated HBIs [13]. This enrichment is consistent with a sea ice origin and is likely derived from *Haslea* and/or *Navicula* species of diatoms, which live in the brine channels at the base of Antarctic sea ice and bloom in spring [12], [13], [14]. In contrast, the lower δ^13^C values of triene and other poly-unsaturated isomers are consistent with a phytoplanktonic origin [13]. Planktonic diatom species that bloom in open waters near the marginal ice zone, such as *Rhizosolenia* spp., are likely responsible for the synthesis of triene and other poly-unsaturated HBI isomers [12], [13], [14]. Based on their occurrence in sediments and their specific origin, these and some other related HBIs markers have been used as proxies for sea ice reconstruction in the Arctic [15], [16], [17] and Antarctic [13], [18]. In particular, the relative abundance of diene to triene (hereafter, the D/T ratio) has been successfully used to estimate the relative contributions of sea-ice-derived and planktonic diatoms to the total primary production in Antarctica [13]. Therefore, HBIs may prove useful for ecological studies at higher trophic levels as ice-based *versus* phytoplankton biomarkers in polar marine ecosystems. Three recent studies have detected HBI isomers in pelagic zooplankton and benthic macrofauna in the Arctic [19], [20], [21], demonstrating that these diatom-specific biomarkers are transferred to Arctic consumers and establishing their potential for determining the relative contribution of ice algae and pelagic phytoplankton to higher trophic levels.

Here, we investigated the potential of this approach for studying the Antarctic pelagic ecosystem. Our first aim was to determine whether HBIs are transferred to key pelagic species in Antarctica. We collected specimens of two krill species, two planktivorous fish species and three seabird species and analysed the presence and concentrations of HBIs. Our second aim was to identify whether the sea-ice-specific diene was more abundant in species preferentially feeding in ice-covered waters (cryopelagic species) than in pelagic species by comparing corresponding tissues among closely related taxa. Finally, we tested (i) whether Antarctic seabirds incorporate more diene during a year of extended pack ice than during a year of reduced ice and (ii) whether a high contribution of ice algal-derived organic matter predict key ecological parameters; i.e., earlier breeding and higher reproductive success.

## Materials and Methods

### 1. Ethics statement

Ethical approval for all procedures was granted by the ethics committee of the Ministère de l'Environnement and the French Polar Research Institute (Institut Paul Emile Victor – IPEV), which approved all our fieldwork. The experiments complied with the Code of Ethics of Animal Experimentation in the Antarctic.

### 2. Study site and specimen collection

**Figure 2:**
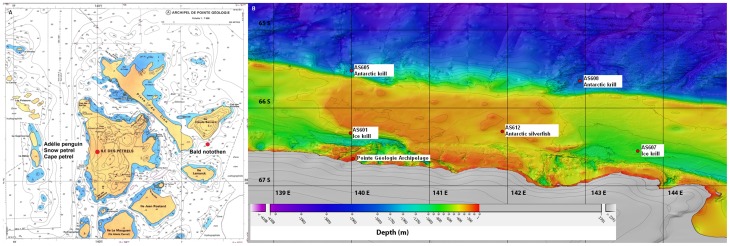
Map showing the location of the Pointe Géologie archipelago and all the sampling sites off Adélie Land, Antarctica. (A) Samples of Adélie penguin, snow petrel and cape petrel were collected on Petrel Island; bald notothen were fished under the sea closed to Bernard Island, Pointe Géologie archipelago. (B) Ice krill, Antarctic krill and Antarctic silverfish were collected off Adélie Land (see the method section for GPS locations). The high-resolution bathymetry of the areas is represented [48].

Table 1 summarises information on specimen collection. Briefly, seabirds (see below) and the bald notothen *Pagothenia borchgrevinki* were studied in the Pointe Géologie Archipelago (Adélie Land, Antarctica, 66°40′S, 140°01′E, Figure 2 A), whereas the ice and Antarctic krill (*Euphausia crystallorophias* and *E. superba*, respectively) and the Antarctic silverfish *Pleuragramma antarcticum* were collected from the Research Icebreaker Astrolabe using an Isaacs-Kidd Midwater Trawl (IKMT). The Adélie Land area is ice covered seasonally, with land-fast ice typically forming in March/April and breaking up in November/December. The cruise was conducted in January 2011 in open waters (between 65°31′S/66°33′S and 139°59′E/143°60′E, Figure 2 B) during the Austral summer.

**Table 1:** Specimen information: common and scientific names of species, stage, tissue, sampling date and site.

Species	Stage	Tissue type	N	Collection date	Sampling site
KRILL					
Antarctic krill *Euphausia superba*	Adult	Whole body	27*	January 2011	Continental slope
Ice krill *Euphausia crystallorophias*	Adult	Whole body	38*	January 2011	Adélie Land coast
FISH					
Antarctic silverfish, *Pleuragramma antarcticum*	Adult	Muscle	6	January 2011	Continental slope
Bald notothen, *Pagothenia borchgrevinki*	Adult	Muscle	9	December 2010	Pointe Géologie Archipelago
SEABIRD					
Adelie penguin, *Pygoscelis adeliae*	Chick	T1	12	Dec 2009–Jan 2010	Petrels island
Adelie penguin, *Pygoscelis adeliae*	Chick	T2	12	Dec 2010–Jan 2011	Petrels island
Adelie penguin, *Pygoscelis adeliae*	Egg	Whole egg	4	December 2011	Petrels island
Snow petrel, *Pagodroma nivea*	Egg	Whole egg	5	December 2010	Petrels island
Snow petrel, *Pagodroma nivea*	Egg	Whole egg	14	December 2007	Petrels island
Snow petrel, *Pagodroma nivea*	Chick	Stomach oil	13	January 2011	Petrels island
Cape petrel, *Daption capense*	Chick	Stomach oil	4	January 2011	Petrels island

* Pool of individuals.

T1: Subcutaneous adipose tissue, retroperitoneal adipose tissue, gastrocnemius muscle, pectoralis muscle, quadriceps muscle, liver, stomach content.

T2: Same tissues than T1, blood and kidney.

For the two euphausiids, oceanic Antarctic krill were collected at two locations over the continental slope (site AS605: 65°31′S, 140°00′E and site AS608: 65°39′S, 142°56′E, Figure 2 B), whereas neritic ice krill [22] were collected at two coastal stations (site AS601: 66°19′S, 139°59′E and site AS607: 66°33′S, 143°40′E, Figure 2 B). Krill exploit both sea ice and open water habitats throughout the year, but larval and postlarval krill are strongly associated with the underneath of the sea ice [23], [24]. The Antarctic silverfish was collected at the site AS612 (66°18′S, 141°56′E°, Figure 2 B) and is the predominant mid-water notothenioid fish in neritic Antarctic waters. During adulthood, it exploits open water habitats; however, juvenile developmental stages and spawning occur under and within sea ice [25], [26]. Bald notothen *Pagothenia borchgrevinki* were sampled under the sea ice closed to Bernard Island (Figure 2 A) using a fishing rod in December 2010. This species is a common cryopelagic fish that forages underneath of the sea ice [26], [27]. For the two fish species, muscle samples were excised.

Pointe Géologie Archipelago is a breeding area for approximately 36,000 pairs of Adélie penguins (*Pygoscelis adeliae*), 870 pairs of snow petrels (*Pagodroma nivea*) and 450 pairs of cape petrels (*Daption capense*) [28]. Seabirds were sampled at Petrels Island, the main island of the Pointe Géologie Archipelago, where the French Antarctic station, Dumont D'Urville is located (Figure 2 A). The snow petrel is confined to high Antarctic waters feeding primarily on fish among ice floes. The cape petrel has a wider latitudinal distribution and feeds primarily on crustaceans in Adélie Land [29], [30], [31]. All procellariids store stomach oil from the breakdown of ingested food and the differential digestion rates of proteins and lipids. We collected both abandoned petrel eggs and the stomach oil of chicks during the 2010/2011 breeding season (Table 1) for HBI analysis. The Adélie penguin is an ice-associated diver that feeds in areas of 20 to 80% ice, but it can also forage in the open sea, under pack ice and under coastal fast ice [32], [33]. Abandoned eggs and dead chicks of Adélie penguins were collected (Table 1) and blood samples taken. The stomach; liver; kidneys; quadriceps, pectoralis and gastrocnemius muscles; and subcutaneous and retroperitoneal adipose tissues were sampled when possible. The sampling dates of chicks (from the 28^th^ of December to the 21^st^ of January) did not differ between 2009/2010 and 2010/2011 (paired t-test, t = −1.874, df = 8, p = 0.098). All samples were immediately frozen at −80°C until analysis at the LOCEAN/IPSL (Paris, France) laboratory.

### 3. HBI analyses

HBI analyses were conducted according to a previously described procedure [15]. Each biological tissue was freeze-dried and an internal standard (7-hexylnonadecane) was added to an aliquot of freeze-dried sample (approximately 1 g dry weight). The sample was saponified using a solution of KOH (4N, MeOH/H2O, 80/20) for 2 hours at 80°C, and the non-saponifiable lipids (NSL) were extracted into hexane (3×10 mL). The extract was purified using open column chromatography (SiO_2_ 50 g.g^−1^ NSL; hexane 8 mL) to yield an apolar lipid fraction containing the HBIs. This hydrocarbon fraction was analysed using an Agilent 7890A gas chromatograph (GC) fitted with a 30 m fused silica Agilent J&C GC column (0.25 mm i.d., 0.25 µm film) coupled to a Agilent 5975C Series mass selective detector (MSD). A ramped oven temperature profile (40–300°C; 10°C/min) followed by an isothermal interval (300°C for 10 min) was used. Data were collected and analysed with Agilent Chemstation software. Individual compounds were identified and quantified using both scan (m/z 50–500 mass range) and selective ion monitoring (SIM) techniques with a constant ionisation potential (70 eV). Mass spectral data were used to identify the occurrence of HBIs, whereas SIM chromatograms were used to quantify the abundances of diene (m/z 348.3), triene (m/z 346.3), tetraene (m/z 344.3) and pentaene (m/z 342.3) by peak area integration. Individual HBI isomers were identified by comparing their GC retention indices and mass spectra with those of previously authenticated standards. HBI relative abundances were calculated based on their individual GC-MS responses and those of the internal standard (7-hexylnonadecane, m/z 266). As we did not determine their individual response factors, HBI abundances were expressed in ng.g^−1^ relative to the internal standard. Procedural blanks were analysed for each 20 samples to ensure the absence of contamination.

### 4. Prey identification in Adélie penguin food samples

In the laboratory, chick stomach contents were placed in a large flat-bottomed tray. Sixteen stomachs contained prey items that were not completely digested. The most abundant prey items were identified to species in three to six sub-samples of each food sample. Prey were identified through examination of exoskeletons (for crustaceans) and of otoliths and bones (for fish). Preys items were counted in each sample. We estimated the proportions of fresh mass represented by each species.

### 5. Sea ice data

Daily remote-sensing sea ice concentration maps, retrieved from the Advanced Microwave Scanning Radiometer–Earth Observing System (AMSR-E), were obtained from the University of Hamburg, Institute of Oceanography from the web site ftp://ftp-projects.zmaw.de/seaice/AMSR-E_ASI_IceConc/no_landmask/hdf/s6250/ [34]. Sea ice concentration data were calculated with the ARTIST sea ice (ASI) concentration algorithm using AMSR-E 89 GHz brightness temperatures. We extracted daily sea-ice concentrations in the area within 137.5°E-142.5°E and 65°S-67°S (approximately 100 km from the Dumont D'Urville station) from September 2007 to March 2011.

### 6. Ecological data

Phenological and reproductive success data were obtained for seabirds via long term monitoring programs conducted by the Centre d'Etudes Biologiques de Chizé [35], [36]. The peak dates of egg laying and hatching, as well as the total numbers of Adélie penguin breeding pairs, chicks in crèche and fledglings were monitored on Petrels Island. For snow petrels, the number of eggs, hatching date and number of fledglings were monitored daily for 282 nests in three colonies located on Petrels Island.

### 7. Statistical analyses

All statistical analyses were performed using R 2.14.2 [37]. It was recently proposed that the D/T ratio reflects the relative contribution of sea ice and planktonic algae to sediments [13], [17]. However, HBIs may degrade or be metabolised while incorporated within tissue. Because of its additional double bond, the triene is likely to degrade much quicker than the diene, we first analysed diene and triene separately, then analysed D/T ratios. Significantly higher D/T ratios were all validated by either a significantly higher diene value and/or a significantly lower triene value. We therefore decided to present the D/T ratio results as a proxy of the relative contribution of sea ice and planktonic derived organic matter to consumer diets. We used Wilcoxon rank sum tests and pairwise Wilcoxon tests with post-hoc Bonferroni corrections to test for differences in D/T ratios between closely related species and years. We used paired t-tests to test for differences between years in sea ice concentrations.

## Results

### 1. Are HBI diatom biomarkers transferred across the Antarctic trophic web?

Diene and triene were detected in all Antarctic pelagic organisms, including krill, fish and seabirds (Table 2). The abundance of diene varied from 0.03 to 16.71 ng.g^−1^ and the abundance of triene varied from 0.53 to 473 ng.g^−1^ both relative to the internal standard. It is important to notice that HBI analyses were conducted either on whole organisms or biological tissues on dissected organisms. The relative abundances of diene and triene were the highest in krill and in stomach contents/oils, livers, eggs of seabirds, whereas diene and triene were in very low abundance in muscles of Adélie penguins and fish, kidneys, adipose tissues, and blood of Adélie penguins. Moreover, concentrations of diene varied greatly between tissue type in Adélie penguin chicks (Kruskal-Wallis χ^2^ = 67.147, df = 8, p<0.001). The highest concentrations of HBIs in Adélie penguin were detected in the stomach content, followed by eggs and liver. The lowest concentrations were found in the quadriceps, pectoralis and gastrocnemius muscles, kidneys, subcutaneous and retroperitoneal white adipose tissues and blood.

**Table 2:** Abundance of diene, triene (mean ± SE, in ng.g^−1^ relative to the internal standard) and the diene/triene ratio in Antarctic pelagic species (whole organism, tissues or eggs) collected during the austral summers 2007/2008, 2009/2010 and 2010/2011.

Species	Diene								Triene								Diene/Triene							
**Krill**					N	2010/2011							N	2010/2011							N	2010/2011		
Ice krill (whole body)					38	11.10	±	2.00					38	273.05	±	20.88					38	0.03	±	0.003
Antarctic krill (whole body)					27	11.49	±	1.17					27	266.13	±	22.94					27	0.04	±	0.003
**Fish**					N	2010/2011							N	2010/2011							N	2010/2011		
Bald notothen (muscle)					9	0.30	±	0.10					9	0.53	±	0.04					9	0.58	±	0.19
Antarctic silverfish (muscle)					6	0.97	±	0.36					6	20.35	±	8.88					6	0.06	±	0.01
**Adélie penguins**	N	2009/2010			N	2010/2011			N	2009/2010			N	2010/2011			N	2009/2010			N	2010/2011		
Egg					4	7.04	±	1.41					4	269.60	±	42.78					4	0.03	±	0.004
Stomach content	12	16.71	±	7.99	12	10.23	±	1.76	12	109.71	±	80.76	12	473.10	±	101.50	12	0.24	±	0.07	12	0.03	±	0.01
Subcutaneous adipose tissue	10	1.42	±	0.92	3	0.24	±	0.03	10	1.56	±	0.39	3	12.59	±	0.63	10	0.3	±	0.08	3	0.02	±	0.003
Retroperitoneal adipose tissue	6	1.47	±	1.35	10	0.23	±	0.07	6	2.47	±	1.49	10	12.05	±	1.49	6	0.27	±	0.13	10	0.02	±	0.003
Liver	10	14.96	±	7.86	13	6.62	±	1.44	10	64.44	±	28.11	13	294.02	±	84.74	10	0.17	±	0.05	13	0.03	±	0.003
Gastrocnemius muscle	5	0.33	±	0.19	12	0.03	±	0.02	5	1.87	±	0.75	12	3.25	±	0.34	5	0.12	±	0.05	12	0.01	±	0.005
Pectoralis	5	0.47	±	0.12	6	0.12	±	0.04	5	2.43	±	1.13	6	5.81	±	0.74	5	0.28	±	0.1	6	0.02	±	0.01
Quadriceps	8	0.71	±	0.29	11	0.11	±	0.04	8	1.74	±	0.62	11	4.42	±	0.55	8	0.32	±	0.1	11	0.03	±	0.01
Kidney					11	0.22	±	0.07					11	5.73	±	0.94					11	0.06	±	0.03
Blood					10	0.45	±	0.09					10	17.11	±	2.95					10	0.03	±	0.01
**Snow petrels**	N	2007/2008			N	2010/2011			N	2007/2008			N	2010/2011			N	2007/2008			N	2010/2011		
Egg	14	0.80	±	0.16	5	3.23	±	1.45	14	20.16	±	3.81	5	284.76	±	125.26	14	0.04	±	0.01	5	0.01	±	0.01
Stomach oil					13	8.67	±	0.41					13	77.69	±	8.64					13	0.12	±	0.01
**Cape petrels**					N	2010/2011							N	2010/2011							N	2010/2011		
Stomach oil					4	1.32	±	0.51					4	80.62	±	23.17					4	0.02	±	0.002

### 2. Do D/T ratios vary among Antarctic consumers?

**Figure 3:**
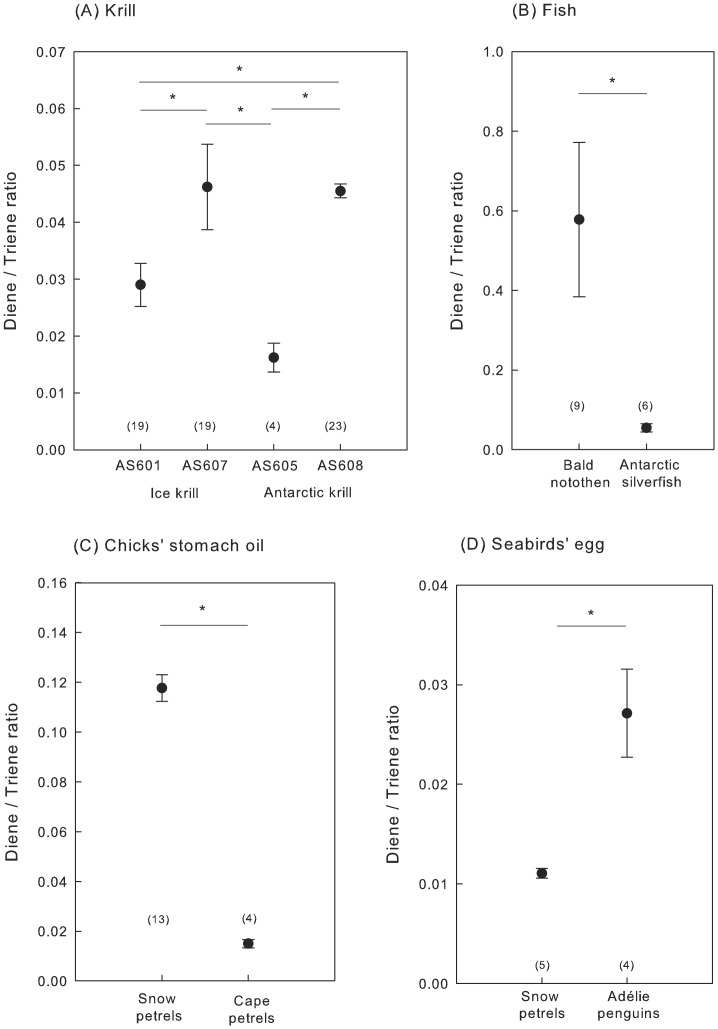
Diene and triene in key marine species from Adélie Land, Antarctica. The D/T ratio (mean ± SE) were compared between (A) two euphausiid species (ice krill and Antarctic krill) at four sampling sites, (B) two fish species (bald notothen and Antarctic silverfish), (C) eggs of two seabird species (snow petrel and Adélie penguin) and (D) chick stomach oil of two seabird species (snow petrel and cape petrel).

D/T ratios did not differ between Antarctic and ice krill but differed significantly between sampling sites but eastern sites (i.e. AS607 and AS608) having significantly higher D/T ratios than western sites (AS601 and AS605, pairwise Wilcoxon tests with post-hoc Bonferroni corrections, p<0.01, Figure 3 A). In contrast, no significant difference was observed between neritic (AS601 and AS607) and pelagic (AS605 and AS608) sampling sites (pairwise Wilcoxon tests with post-hoc Bonferroni corrections, p<0.210).

D/T ratios were significantly higher in muscles of bald notothen from under the ice than in muscles of Antarctic silverfish collected in open waters (Wilcoxon rank sum test: N = 15, W = 51, p = 0.003, Figure 3 B).

D/T ratios in chick stomach oil were significantly higher in snow petrels than in cape petrels (Wilcoxon rank sum test: W = 0, p = 0.004, Figure 3 C). In eggs, D/T ratios were significantly higher in Adélie penguins than in snow petrels (Wilcoxon rank sum test: N = 9, W = 20, p = 0.016, Figure 3 D).

### 3. Do D/T ratios in Antarctic consumers vary with sea ice conditions?

**Figure 4:**
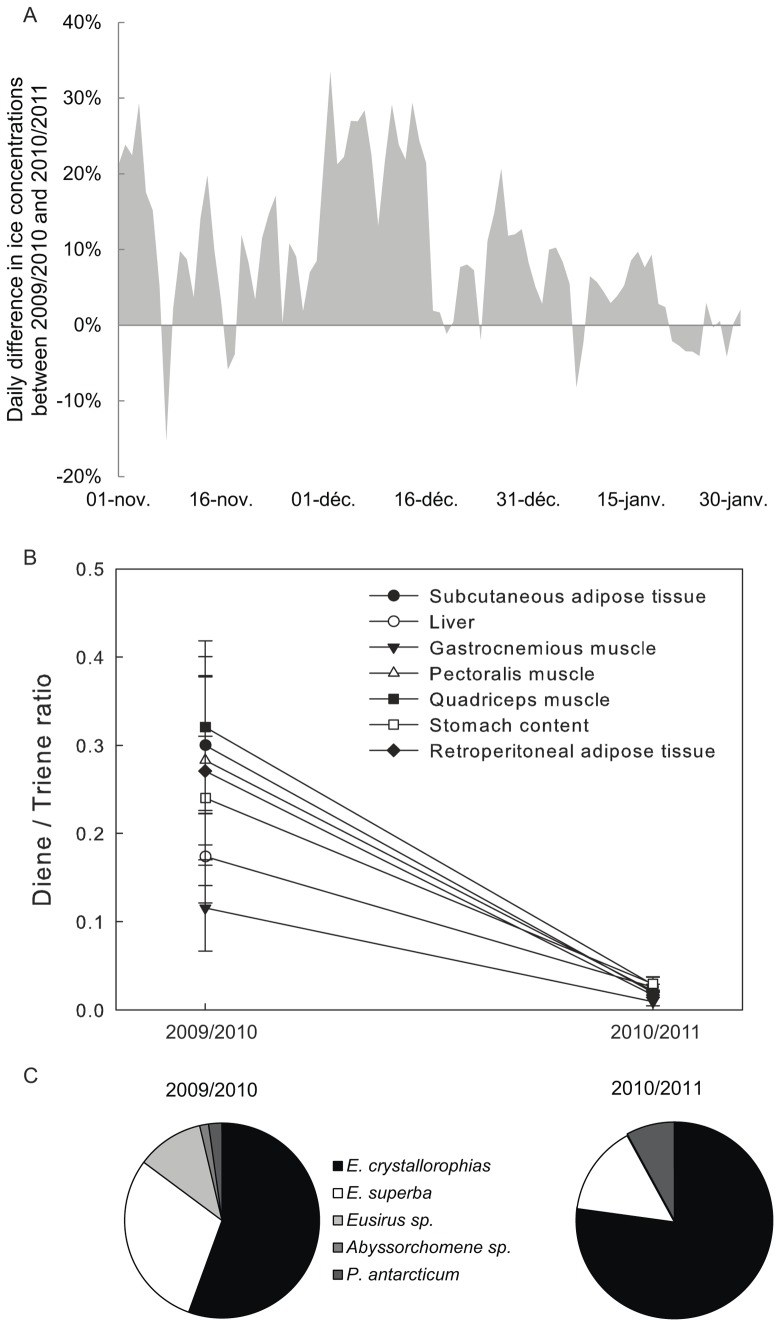
Differences in ice concentrations, HBIs biomarker concentrations and prey characteristics of Adélie penguin chicks between 2009/2010 and 2010/2011. (A) Daily difference in sea ice concentration from November to January between the 2009/2010 and 2010/2011 breeding season, (B) D/T ratios (mean ± SE) measured in tissues and stomach contents of Adélie penguin chicks, (C) prey composition and abundance in chick stomach contents.

During the Adélie penguin breeding season (defined as the period from the onset of egg-laying, around the 1^st^ of November until the start of chicks' fledgling, the 31^st^ of January), sea ice concentrations were significantly higher in 2009/2010 (mean ± SE during these 3 months: 55.8%±2.1%) than in 2010/2011 (46.3%±1.9%, paired t-test, t = 9.086, df = 91, p<0.001, Figure 4 A). Adélie penguin dead chicks were collected from the 28^th^ of December (just after hatching) to the 21^st^ of January (30-day old). During this sampling period, sea ice concentrations were also significantly higher in 2009/2010 (37.2%±1.2%) than in 2010/2011 (31.5%±1.2%, paired t-test, t = 5.810, df = 24, p<0.001).

D/T ratios in all analysed tissues (liver, quadriceps, pectoralis and gastrocnemius muscles, subcutaneous and retroperitoneal white adipose tissue) and in stomach contents of Adélie penguin chicks were significantly higher during the 2009/2010 breeding season than during the 2010/2011 breeding season (p<0.001 for all Wilcoxon tests; Figure 4 B).

Prey identified in stomach contents revealed less consumption of ice krill (Wilcoxon test, W = 6, p = 0.015) and more consumption of Antarctic krill (Wilcoxon test, W = 44.5, p = 0.044) in 2009/2010 than in 2010/2011 (Figure 4 C). The mean biomass of Antarctic silverfish in stomach contents did not differ significantly between years (Wilcoxon test, W = 13, p = 0.104, Figure 4 C).

The long term monitoring programme conducted on Petrels Island, where Adélie penguin dead chicks were collected, revealed that the total number of Adélie penguin pairs that bred did not differ significantly between years (15999 breeding pairs in 2009/2010 and 14694 in 2010/2011). However, both the number of chicks in crèche (N = 15624 and N = 9561, respectively) and the number of fledglings (N = 15405 and N = 9256, respectively) were higher in 2009/2010 than in 2010/2011. Egg-laying date (peak: 17/11/2009 and 22–23/11/2010) and hatching date (peak: 21/12/2009 and 23–24/12/2010) were approximately 2.5 days earlier in 2009/2010 than in 2010/2011.

We investigated HBI concentrations in abandoned snow petrel eggs between 2007 and 2010. Female snow petrels forage at sea from mid-November to mid-December while forming their single eggs. Analysis of satellite data revealed that sea ice concentrations were significantly higher in 2007 than in 2010 during this period (t = 5.647, df = 28, p<0.001, Table 3). D/T ratios of eggs were also significantly higher in 2007 than in 2010 (Wilcoxon rank sum test, N = 19, W = 70, p<0.001, Table 3). Among the 282 monitored nests, both the number of breeding pairs and the number of fledglings were higher in 2007/2008 than in 2010/2011; however, the mean hatching date was 2 days later in 2007/2008 than in 2010/2011 (Table 3).

**Table 3:** Variation in sea ice concentration, D/T ratios in snow petrel eggs and breeding parameters (number of breeding pairs, number of fledglings and hatching date) of snow petrels on Ile des Pétrels, Adélie Land.

Breeding Season	2007/2008	2010/2011
Sea ice concentration (%)	65.3±2.2	54.1±2.6
D/T ratio in eggs	0.04±0.01	0.01±0.0005
Number of breeding pairs	204	193
Number of fledglings	139	100
Hatching date (January)	21.42±0.25	19.42±0.20

## Discussion

### 1. Are HBI diatom biomarkers transferred across the Antarctic trophic web?

HBI markers have recently been identified in Arctic benthic macrofauna and pelagic zooplankton [19], [20], [21], but their applicability to Antarctic marine species has been largely unexplored. The present study provides the first evidence that diatom HBI biomarkers are transferred across an Antarctic pelagic ecosystem up to higher trophic levels. Specifically, the occurrence of the sea ice biomarker diene may provide a new way of assessing the contribution of ice algal-derived organic matter in the diet of consumers, given this HBI lipid was detected in all organisms investigated in this study.

In Adélie penguin chicks, we observed large differences in the concentrations of HBIs between tissue types, suggesting a time-integrated assimilation of HBI lipids within organisms as for fatty acids [e.g. 38]. Our results suggest that the liver preferentially accumulates HBIs relative to adipose tissue, muscle and kidneys which might be useful for assessing the importance of sea ice primary production to vertebrate species over long periods. D/T ratios in stomach contents may permit estimation of the consumption of cryopelagic versus pelagic prey over the preceding days. The presence of HBIs in eggs indicates that these compounds are transferred from females to their eggs. However, little is known regarding the mechanisms of HBI incorporation into tissues, including assimilation, metabolic and elimination rates.

### 2. Do D/T ratios vary among Antarctic consumers?

Krill use both ice and pelagic food sources throughout the year [39], [40]. As ice krill prefer more neritic regions relative to Antarctic krill [22], we expected higher abundances of diene, lower abundances of triene, or both in ice krill. Unexpectedly, the two species did not differ in their D/T ratios, suggesting a similar consumption of cryopelagic and pelagic diatoms in Adélie Land during the summer. However diene and triene abundances differed greatly between sampling sites, with eastern sites having significantly higher D/T ratios than western sites. These results suggest that the incorporation of HBIs in krill swarms may be related to the presence of drifting ice flows. In summer, high densities of Antarctic krill in the 0–2 m surface layer have been documented in the under-ice habitat but not in the open surface layer [24]. In Adélie Land, fast ice broke up in spring, but drift ice was still present during krill sampling. An alternative, non-mutually exclusive hypothesis is that krill swarms vary in the relative proportion of sub-adults to adults between sampling sites [41]. As younger stages are more strongly associated with sea ice, D/T ratios may increase with increasing occurrence of juveniles and sub-adults in krill swarms.

D/T ratios were higher in muscles of bald notothen collected under the ice than in muscles of Antarctic silverfish collected in open waters. The bald notothen feeds primarily on cryopelagic copepods, pteropods, gammarids and hyperiids in platelet ice and water beneath the ice [26], [27], whereas the Antarctic silverfish feeds on euphausiids, pelagic copepods, gastropods and gammarids [25]. Therefore, the D/T ratio in muscle appears to be a good proxy of the contribution of ice algal-derived organic matter in the diet of fish species. We also detected diene in adult Antarctic silverfish. This result may reflect the consumption of partially ice-associated prey or the persistence of diene from juvenile stages, which are associated with sea ice and actively feed on sympagic zooplankton [26], [42]. Therefore, even at low levels, the occurrence of diene indicates an interaction with sea ice.

At the top of the trophic web, D/T ratios within chick stomach oil were higher in snow petrels, which are piscivorous seabirds confined to the pack ice zone [29], [30], [31], [32], than in cape petrels, which are krill consumers exploiting open inshore waters [29]. Thus, our findings are consistent with a higher consumption of ice-associated prey in snow petrels than in cape petrels. Moreover, D/T ratios were higher in the eggs of Adélie penguins than in those of snow petrels, suggesting that female Adélie penguins rely more heavily on ice-associated prey during egg formation than snow petrels. As ice has yet to break up during the period of energy accumulation for egg building, it is possible that cryopelagic prey are more readily accessible to divers, such as penguins, than to aerial foragers, such as petrels.

### 3. Are D/T ratios in Antarctic consumers related to sea ice conditions?

To control for among-tissue differences, we performed comparisons on the same tissue types of Adélie penguins during the two consecutive breeding seasons. D/T ratios in all tissues and stomach contents of Adélie penguin chicks were lower in 2010/2011 than in 2009/2010, coinciding with 10% lower daily sea ice concentrations during the 2010/2011 breeding season than in the 2009/2010 breeding season. Although little is known about HBI production and D/T ratio changes through time in Antarctica, these results suggest a reduced contribution of ice algal-derived organic matter in the diet of chick-rearing Adélie penguins in 2010/2011. Furthermore, prey items in chick stomachs confirmed a change in diet between years. Although ice krill was the predominant prey (as reported elsewhere; e.g., [43]), Antarctic krill were more abundant during 2009/2010, the period of greater sea ice concentration. Extensive sea ice induces high overall primary production and the dominance of ‘large’ diatoms over ‘small’ cryptophytes [44]. As Antarctic krill are more efficient at grazing large particles [23], the survival and recruitment success of Antarctic krill are enhanced during years of heavier ice conditions [45], [46]. In turn, Adélie penguins rely more heavily on Antarctic krill, breed earlier and have higher breeding success, as reported here and in previous studies [42], [47]. Similarly, lower D/T ratios were reported in snow petrel eggs during a year of reduced sea ice concentration and were accompanied by high breeding failure.

Annual variation in the phenology and ecology of Antarctic top predators with variation in the extent of sea ice has been widely discussed [47]. However, the importance of ice-related primary production has been little studied due to the lack of appropriate tools. HBI lipid biomarkers are a promising tool for tracking carbon flow in ice-covered oceans. As many migratory species feed on the highly productive, ice-covered waters of the Southern Ocean [6], the application of HBI lipid measures to the study of sea ice primary production may be extended to larger spatial scales. Ultimately, they may be useful for understanding the consequences of shrinking sea ice on the structure, functioning and dynamics of polar marine trophic webs.
